# New Jersey: A Case Study of the Reduction in Urban and Suburban Air Pollution from the 1950s to 2010

**DOI:** 10.1289/ehp.1103540

**Published:** 2011-05-27

**Authors:** Paul J. Lioy, Panos G. Georgopoulos

**Affiliations:** Environmental and Occupational Health Sciences Institute (EOHSI), University of Medicine and Dentistry of New Jersey (UMDNJ)–Robert Wood Johnson Medical School and Rutgers University, Piscataway, New Jersey, USA

**Keywords:** air pollution, Clean Air Act, control strategy, criteria air pollutants, history, NAAQS, New Jersey, ozone, PM, sprawl

## Abstract

Background: Air pollution has been a topic of intense concern and study for hundreds of years. During the second half of the 20th century, the United States implemented regulations and controls to reduce the levels of criteria air pollutants and achieve the National Ambient Air Quality Standards (NAAQS) for the protection of human health, while concurrently reducing the levels of toxic air pollutants.

Objective: In this commentary we trace the changes in air pollution in New Jersey as a case study, demonstrating the impact of local, state, and federal strategies to control emissions of pollutants and pollutant precursors from the 1950s until today.

Discussion: The original NAAQS (1970–1995) have been achieved, and significant progress has been made to achieve revised standards for ozone and particulate matter (PM) < 2.5 µm in aerodynamic diameter (PM_2.5_) in New Jersey, which in the past was considered a highly polluted industrial state.

Conclusions: Assuming no reversals on current regulations because of some major event or energy infrastructure disruption, outdoor air pollution reductions will continue to address health risks among specific segments of the general population affected by ozone/PM and pollution caused by neighborhood, local, and regional point and mobile sources.

Air pollution problems can be traced to the development of industrialized and urbanized locales over many centuries. Systematic efforts to control air pollution and concurrently protect public health commenced mostly during the second half of the 20th century, intensifying since the 1960s (Reitze 1999; Stern 1962; Vallero 2008). Although national assessments of air pollution trends are available [e.g., U.S. Environmental Protection Agency (EPA) 2010b], very useful insights can be gained by considering the history of air pollution in New Jersey as a case study. In this commentary we focus on how air pollution has decreased in intensity and changed in character in New Jersey over the past 50–60 years, and we address some of the issues and challenges ahead.

## Background

Established public perception associates New Jersey, the most densely populated state in the nation, with industrial emissions and high air pollution levels. Although there was a certain truth to this perception in the first half of the 20th century, substantial improvements have taken place over the past 50–60 years. In fact, data on the progress in reducing New Jersey air pollution are documented in the New Jersey Department of Environmental Protection (NJDEP) annual reports, published since 1971 (NJDEP 2011). The efforts toward clean air have not been a linear or simple process, because many factors had to be considered, including the combined impacts of industrial emissions and suburban lifestyles. Changes in lifestyle have resulted in increased automobile traffic and vehicular miles traveled (VMT) (Gutt et al. 2000; Salmore and Salmore 2008), affecting the character, patterns, and intensity of air pollution emissions, transport, and accumulation. In contrast to most states with ~ 9 million residents, New Jersey has no city with a population approaching even one-half million. In addition, the state’s small size (7,417 mi^2^) and its high population density (1,110/mi^2^) mean that mobile and stationary sources are generally located in proximity to populated areas (Salmore and Salmore 2008).

As in New York City, New York, and Pittsburgh, Pennsylvania, attempts to control air pollution in New Jersey started prior to the formation of the U.S. EPA in 1970 (Beck 2007). For instance, Hudson County implemented a smoke control act in 1931 (Woodward 1955). The approaches considered for addressing problems ranged from the rational to the ridiculous (Mallette 1957a, 1957b). As an example of the latter, in one New Jersey county a proposal was implemented to mask hydrogen sulfide odors (rotten egg–like) emitted by a chemical plant by using a 400-lb drum that released a deodorizer at times of high odor (Mallette 1957c). In 1954, New Jersey adopted one of the first statewide air pollution laws, the Air Pollution Control Act (State of New Jersey 1954), which established an Air Pollution Control Commission and defined the relationships between state and local pollution control organizations. The New Jersey act required control strategies for open burning (code effective May 1956), incineration, and coal combustion (NJDEP 2000, 2002, 2006).

Significant amendments to the New Jersey 1954 Air Pollution Control Act were passed in 1967, and these regulations have continued to strengthen over subsequent decades. These regulations gave the state the ability to set ambient standards, form a cabinet agency to regulate pollutants, set new source performance standards, and control sulfur content in fuels (Reitze 1999; Salmore and Salmore 2008; State of New Jersey 1954). The 1970 Clean Air Act (CAA) gave the newly formed U.S. EPA the primary role in developing National Ambient Air Quality Standards (NAAQS) and implementing national emission regulations and control strategies (U.S. EPA 2010a). A review article by Bachmann (2007) provides an excellent summary of the problems and progress made in implementing the CAA and the influence of amendments passed by Congress in 1977 and 1990.

The New Jersey legislature created the NJDEP on 22 April 1970, coinciding with America’s first official Earth Day, and adopted over 200 environment-related measures between 1970 and 1975. Changes to administrative codes established ambient air quality standards; control and prohibition of particle and gas emissions; control of smoke; permitting of facilities; prevention of toxic air pollution, landfill emissions, automobile vapor and combustion emissions, and truck and shipping emissions; reductions in chemical storage facilities; and so on. More recent regulations deal with population-based emission sources such as architectural coatings and consumer products.

**Figure 1:**
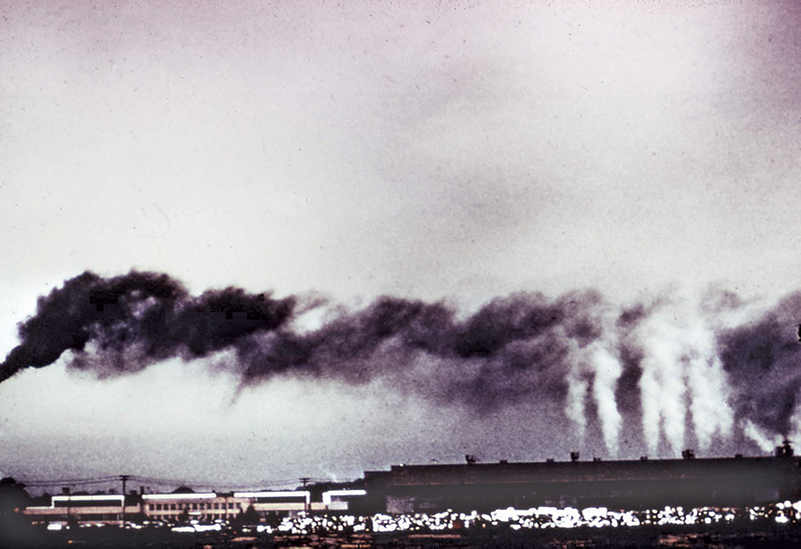
Historical air pollution in New Jersey (source: George and Carol Wolff; from presentation for Air Pollution Control Association, Middle Atlantic States Section, Newark, NJ, circa 1974).

Figure 1 provides a historical picture of New Jersey air pollution emitted by stationary sources during the 1950s in the industrial corridor just west of Manhattan and Staten Island, New York. The corridor included Paterson, a textile manufacturing center; Passaic, an industrial center that included a vinyl chloride plant; industrial sections of Newark and Elizabeth; the Standard Oil refinery in Linden; and the site of a metal (lead) smelting facility that operated from the 1880s through 1982. Coincidently, local electrical generation facilities (coal, nuclear, etc.) and natural gas (first derived from coal gasification plants) were introduced over time. Other point sources were located throughout the state, including commercial and industrial activities in Camden, Toms River, Sparta, and Hudson County, New Jersey.

The first train line was built in New Jersey in the 19th century, and trains that were initially operated by steam engines were eventually replaced by electric and diesel engines (Salmore and Salmore 2008). Roadways that traverse the state include the New Jersey Turnpike (northeast to southwest), finished in 1951; the Garden State Parkway (north to south), finished in 1955; and several interstate highway system routes that also began construction in New Jersey in the 1950s (Salmore and Salmore 2008). All of these highways have expanded: The New Jersey Turnpike now has 12–14 lanes in the northern section. These road expansions have led to increases in automobile traffic and emissions and facilitated a major redistribution of the population throughout the state.

Space heating, an area source of wintertime air pollution, moved from using coal, to oil, and then to gas and electricity as preferred fuels. The goal was to reduce sulfur dioxide (SO_2_) and particle emissions into the atmosphere. This occurred at the same time air conditioning became common throughout the state. The latter substantially increased the amount of summertime electrical energy produced locally or imported from coal-burning states to the south and west of New Jersey. The use of air conditioning also increased summertime ambient levels of fine particles containing SO_2_.

Demographic changes over the past 40–60 years have also influenced air pollution in New Jersey. For example, before 1960 New Jersey had three major sectors: agricultural, urban/industrial, and rural (including forests/pinelands). Beginning in the late 1950s, people began to move from the urban areas into what is now characterized as the fourth major sector, the “sprawling suburbs.” This led to reductions of farmland (in the 1950s one-third of active farms disappeared and agricultural land was reduced by 7%) in favor of residential developments. This process continued through the turn of the century (Salmore and Salmore 2008). For each suburban community, new roads and other infrastructure were constructed to support typical residential activities (e.g., schools, commercial facilities, entertainment). The suburbanization of the state also led to changes in transportation routes and the number and types of commercial and service businesses established to support living in and commuting from the suburbs, including suburban malls, which have increased in size, type, and number since the first was built in Bergen County (Salmore and Salmore 2008). The number of VMT per day within and through New Jersey increased as activities relocated to the suburbs. In parallel, heavy industry in New Jersey decreased, and many former industrial locations were eventually reclaimed (e.g., brownfields) for use as residential areas and commercial centers (Salmore and Salmore 2008). For example, the Hudson River waterfront in Hoboken and Jersey City became residences for people traveling to and from the financial districts of New York City.

## Changes in Air Pollution in New Jersey

Air pollution in New Jersey, prior to suburban sprawl, was dominated by pollutants emitted from industrial sources, energy production, and space heating. These air pollutants included SO_2_, soot, total suspended particles (TSP), carbon monoxide (CO), and volatile organic compounds (VOCs). Many of the same pollutants caused acute health-related air pollution episodes (e.g., the London Smog, Donora, PA) and persistent air pollution (e.g., Pittsburgh, PA) (Beck 2007; Bell et al. 2004; Dockery and Pope 1994). Stern (1957) summarized national TSP measurements from 1953 to 1957 using U.S. National Air Sampling Network data. In urban areas TSP was high, with a mean of about 140 µg/m^3^, and the 95th percentile for all the data was > 370 µg/m^3^ (Stern 1957). New Jersey was within this distribution of high levels of TSP.

Annual reporting of New Jersey air pollution began in the 1960s and, with the formation of the U.S. EPA, measurements expanded in both quantity and quality. During the 1950s, one of the main approaches used to identify point source air pollution was a simple Ringelmann Chart of “blackness” of emissions (Stern 1962). One of the first long-term air pollution records for New Jersey is that of the indicator of black smoke called coefficient of haze (COH) (NJDEP 2011). From 1967 to 2007, there was a 10× reduction in the annual COH values, indicating major decreases in uncontrolled combustion source emissions, elimination of residential coal burning, and reduction of sulfur in oil (Vallero 2008). In fact, the largest percentage reduction in COH occurred before the implementation of the 1970 CAA (NJDEP 2011). COH is still measured today, but it is no longer a robust indicator of particulate matter (PM), because black soot has given way to water-soluble sulfate and organic compounds as the dominant components of fine particles < 2.5 µm in aerodynamic diameter (PM_2.5_).

**Figure 2:**
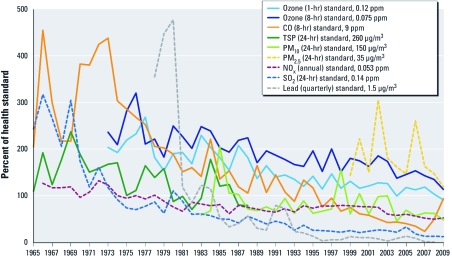
Overall trend for all the criteria air pollutants in New Jersey, 1965–2009, for the annual maximum measured at all monitoring sites in the state shown as a percentage of a pollutant’s level above or below the NAAQS. Historical New Jersey air monitoring data prior to 1975 was received from C. Pietarinen (personal communication) and other data from AirData (U.S. EPA 2011).

The clear downward trend in annual maximum concentrations of criteria air pollutants measured at all sites in New Jersey from 1965 to 2009, reported as the percentage of each pollutant’s levels above or below a corresponding NAAQS, is shown in Figure 2. During the 1960s and 1970s, levels of all measured pollutants were very high, but they began to decline after the 1967 revisions to the New Jersey air pollution code. These revisions took place after the 1966 Thanksgiving New York City SO_2_ and PM episode, which was estimated to have shortened the lives of 366 people in New York City (Schimmel 1978).

During the 1980s, with the exception of ozone, ambient pollutant levels decreased steadily. For example, in 1975, the 8-hr standard (for the three nonoverlapping periods during each day) for CO was exceeded 265 times in Morristown and 100 times at two other urban New Jersey sites. By 1995, there were no exceedances of the CO standard, and there have been none since. This was due to changes in the design of the automobile engine, state inspection and maintenance programs, and alterations in the composition of gasoline used to power automobiles (NJDEP 2011).

Trends in PM levels are a bit more confusing because based on epidemiological studies, there have been changes in the particle size ranges of interest and in permissible exposure levels (annual or 24-hr average) (NJDEP 2011). Early measurements of TSP were replaced by measurements of the PM size fraction < 10 µm in aerodynamic diameter (PM_10_) in the 1980s, which were supplemented in 1999 by measurements of PM_2.5_, determined to be the particles of concern for acute and long-term health effects (Bachmann 2007). Figure 2 indicates that TSP and PM_10_ levels have decreased steadily. PM_2.5_ has a much shorter history of measurements; however, the 24-hr PM_2.5_ standard of 35 µg/m^3^ is rarely exceeded in New Jersey, and the current annual standard of 15 µg/m^3^ (not shown) was not exceeded in 2010 (NJDEP 2011). In the future the PM_2.5_ standard may continue to tighten, but even now, with a continued decrease in levels of PM_2.5_, a suggested 13-µg/m^3^ annual standard would be violated by < 1 µg/m^3^ (NJDEP 2011).

The greatly expanded use of the automobile on the state and national highway systems in the 1960s also led to a new type of air pollution in New Jersey—photochemical smog—a major component of which is ozone. Ozone is formed in the atmosphere as a result of photochemical reactions involving VOCs and nitrogen oxides (NO_x_), which constitute the precursor emissions to secondary pollutants. Photochemical smog was first identified in the 1950s in southern California, but the significance of large-scale atmospheric ozone formation and transport into the mid-Atlantic and northeastern states, including New Jersey, was not understood until the 1970s (Georgopoulos 1995). The regional character of ozone levels in New Jersey, first recognized by Wolff and Lioy (1980), critically limited the effectiveness of local reductions of the precursor emissions for ozone and other oxidants.

New Jersey started to measure ozone specifically in 1973, and levels were above the NAAQS statewide by the mid-1970s (NJDEP 2011). However, the levels could have been higher at earlier periods, because no measuring devices were available to adequately measure ozone (NJDEP 2011). Today, photochemical smog persists statewide, primarily because of increased use of automobiles. Further, individuals living in near-roadway locations can be exposed to a variety of other automobile- and truck-related pollutants and air toxics (Karner et al. 2010).

Once the measurements of ozone started in 1973, the results immediately showed that statewide levels were significantly above the 1-hr NAAQS of 120 ppb promulgated in 1978. This standard was a relaxation, by the Carter administration, from the original 1971 NAAQS of 1 hr at 80 ppb. Based on the original standard, a further review of the data showed that New Jersey had 399 hourly ozone violations in 1978 (NJDEP 2011).The levels have decreased significantly over the past 30 years, and the retired 120-ppb 1-hr standard was achieved statewide in 2004 (Figure 2). The decline in ozone has been due to improvements in the automobile engine and changes in gasoline composition that have reduced hydrocarbon and NO_x_ emissions from mobile sources and reactive hydrocarbons from stationary sources (Georgopoulos 1995). The reduction of the Reid vapor pressure (volatility) of gasoline before the 1989 ozone season led to an immediate decline in the number of violations of the 1-hr standard, from 45 days in 1988 to 18 days in 1989 (NJDEP 1998). After the promulgation of the 80 ppb 8-hr standard in 2002, the number of violations continued to decline, and by 2009 there was only 1 day with an 8-hr concentration > 80 ppb in New Jersey. However, the 8-hr standard was reduced to 75 ppb, and in 2009 the state had 35 days above that level; these occurred at 11 of the 14 operating monitoring sites (NJDEP 2011). Thus, although ozone has persisted as a problem in New Jersey, significant progress has been made in controlling this regional air pollutant, and most residents are now protected from deleterious effects. Efforts to achieve the tightened ozone standard will help to protect more sensitive subgroups of the population (NJDEP 2011).

Ozone remains an issue because emission and transport of ozone precursors (VOCs and NO_x_) and formation/transport of ozone takes place across multiple spatial scales, from regional to local; thus, reductions in the emission of precursors require coordinated actions by multiple states. In addition, although levels of VOC emissions have decreased, NO_x_ continues to provide a reservoir of precursors available for ozone production.

The reductions in ambient ozone levels associated with the control of VOC and NO_x_ from motor vehicles have been offset in the Northeast, and probably other parts of the country, by the continued increase in local (sprawl) and interstate vehicular miles. Since the 1950s, the number of VMT per day has increased throughout the United States and New Jersey; the number of miles traveled per year has increased by > 50% since 1975 (Gutt et al. 2000; Schwemberger et al. 2005). As of 2008, > 200 million miles were traveled per day in New Jersey (NJDEP 2009). Concurrently, changes in automobile engine and exhaust systems [implemented by the U.S. EPA and checked through inspection and maintenance programs (NJDEP 2006)] and in the composition of gasoline (including seasonal differences to reduce photochemically reactive emissions during the summer), along with turnover to newer vehicles, has allowed reductions in ozone to continue in spite of the ever-increasing number of VMT per day. The introduction of electric vehicles is expected to further reduce local VOCs but may increase regional NO_x_ because of increased use of electricity from coal and other energy sources to recharge batteries. Ozone levels may also be affected by increased use of ethanol (E15), a compound that can contribute to ozone production (Ginnebaugh et al. 2010).

Emission factors were required by the CAA, including the continually updated AP-42 document (U.S. EPA 1995), and emissions are tracked using the National Emissions Inventory (U.S. EPA 2009). In some instances, emission control requirements led to the elimination of certain chemical and manufacturing industries in New Jersey, and significant reductions were required of those that remained. Further, restrictions on the levels of sulfur in fuels led to reductions in SO_2_ levels in the atmosphere (NJDEP 2011), and increased use of natural gas for domestic space heating also helped accelerate reductions in ambient levels of SO_2_. The range of observed annual peak values was quite wide until 1981, but since then the variation in measured SO_2_ has been small (Figure 2).

The NAAQS for lead (1.5 µg/m^3^ quarterly) was promulgated in 1978, but atmospheric levels had begun to decline before that time (Bachmann 2007). This coincided with the changeover to unleaded gasoline, because lead in the gasoline poisoned the platinum catalyst used in the catalytic converter to control automobile hydrocarbon and CO emissions. The result was a precipitous drop in atmospheric lead levels that was accompanied by a significant drop in blood lead levels in children and adults; this drop was the first effective use of a biomarker of internal exposure to demonstrate health-related accountability for a national air pollution source (Schwemberger et al. 2005). By 2006, atmospheric lead values in New Jersey were < 0.15 µg/m^3^ quarterly, the new NAAQS adopted in 2008 for lead.

An important consideration for tracking progress in reducing New Jersey’s air pollution is that the bar for achieving cleaner air has been raised for some criteria pollutants, including ozone and PM_2.5_, to protect subpopulations at greater risk of adverse health outcomes. Although the air in New Jersey is getting closer to compliance, and levels should be considered acceptable for a vast majority of the population, the state must now achieve the tighter NAAQS to protect susceptible subpopulations (e.g., children, elderly, individuals with health problems).

**Figure 3:**
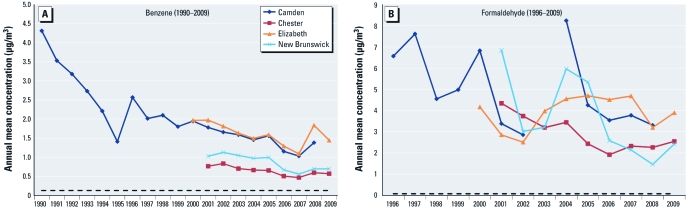
Monitored concentrations of benzene (1990–2009; *A*) and formaldehyde (1996–2009; *B*) in four New Jersey cities. Levels of both pollutants have generally decreased over the past several years (NJDEP 2011).

*Toxic air pollutant levels.* The number of measured air pollutants has expanded to include toxic substances such as benzene, which is released from both the automobile (fuel and exhaust) and industrial/commercial  sources (solvent or raw material) (Vallero 2008), and formaldehyde, which is emitted directly from industrial and combustion sources and is produced indirectly through ambient formation in summertime photochemical smog. Consistent with the implementation of new pollution controls, changes in motor vehicle fuel type and quality, and changes in chemical processes, the levels of each pollutant have decreased between approximately 4× for benzene and approximately 10× for formaldehyde over the past 15–20 years (Figure 3) (NJDEP 2011). However, the levels  for each are still substantially above the 1 in 1-million excess cancer risk benchmark (Caldwell et al. 1998), which means that reductions of emissions are still needed in the future. In addition, although reductions of air toxics emissions are focused on processes and sources that affect ambient air, many pollutants, including benzene and formaldehyde, are also emitted or produced from indoor sources. Therefore, future air toxics controls or prevention strategies (e.g., product replacement) may need to account for indoor sources of pollutants as well (Lioy 2010).

*Impact of regulations and strategies.* Tracking annual maximum pollutant concentrations from the levels observed at all New Jersey monitoring sites relative to historical NAAQS standards (Figure 2) is just one of many possible ways of reporting air quality trends, but this approach provides strong evidence in support of the overall success of state and federal policies in reducing air pollution in New Jersey. In all cases, ambient pollution levels have been reduced to the point where they have either achieved the original NAAQS or they approach current standards. This progress has meant that the vast majority of the public is now protected within a margin of safety from the deleterious effects of criteria pollutants. The changes to the NAAQS for ozone, lead, and PM (now PM_2.5_) have focused attention on small but highly vulnerable subgroups of the population (Bachmann 2007) based on new evidence about health effects in high-risk groups. Therefore, although the air has not been degrading at any location in the state, the targets have become tighter and require further pollution reduction strategies and monitoring, particularly in areas where there are populations at risk. Nonetheless, after > 50 years, New Jersey has made great strides in reducing air pollution levels experienced by the population as a whole.

## Challenges Ahead

All the original NAAQS have been attained in New Jersey, and the recently revised NAAQS for ozone and PM_2.5_ are close to being achieved. However, as in other states, there remain areas with local air pollution problems; these “hot spots” are, in some cases, associated with environmental justice issues. Furthermore, variability in local and regional meteorology may still result, although more rarely, in situations where pollution levels become high. Future efforts should include the use of new tools, such as saturation monitoring in areas of concern, to establish a baseline for determining the strength of hypotheses on the relationships among individual or population exposures within hot spots of point or area source emissions and health outcomes (Zhu et al. 2008). An example is near-roadway air pollution problems (Karner et al. 2010). This has been a concern for years, but future monitoring and exposure–health effects studies must consider the gradients of pollution levels (e.g., aldehydes, particles, noise) from the road to the adjacent populations. Future air pollution reduction strategies will also need to consider *a*) populations at risk because of biologically based susceptibility, age, and so on; *b*) fuels used for energy production; *c*) indoor air pollution; and *d*) consumer and personal products with the same pollutants as outdoor air (Bachmann 2007; Chow et al. 2007; Karner et al. 2010; Mitchell et al. 2007; Nazarenko et al. 2010; Zhu et al. 2008).
